# The impact of beverage consumption on chronic renal failure risk and the mediation of serum metabolites: based on Mendelian randomization study

**DOI:** 10.1186/s12263-025-00773-w

**Published:** 2025-07-11

**Authors:** Zhengshu Wei, Dunsheng Mo, Wenxin Lü, Shangxin Wu, Yan Zhang, Zhen Tang, Yongyi Fan

**Affiliations:** Liuzhou Worker’s Hospital, The Fourth Affiliated Hospital of Guangxi Medical University, Guangxi, 545005 China

**Keywords:** Coffee, Alcohol, Tea, Chronic renal failure, Mendelian randomization, Metabolites

## Abstract

**Background:**

Chronic renal failure (CRF), the end-stage of chronic kidney disease, affects approximately 10% of the global population. While associations between beverage consumption and renal function have been reported, their causal relationships remain unclear. This study aimed to investigate the causal relationships between different beverage consumption and CRF, as well as the mediating effects of serum metabolites.

**Methods:**

Using a two-sample Mendelian randomization (MR) approach, we analyzed genetic data from the UK Biobank and GWAS databases. We examined bidirectional causal relationships between water, coffee, tea, and alcohol consumption with CRF, and screened metabolites significantly associated with CRF from 1,400 metabolites for mediation analysis. Additionally, we evaluated the mediating effects of these metabolites in the relationship between beverage consumption and CRF.

**Results:**

MR analysis showed evidence for a causal association between tea consumption and reduced CRF risk (OR = 0.314, 95% CI: 0.155–0.634, *p* = 0.001), while alcohol consumption was causally associated with increased CRF risk (OR = 1.275, 95% CI: 1.046–1.553, *p* = 0.016). Water and coffee consumption showed no significant associations with CRF. Further analysis identified 11 metabolites significantly associated with CRF. Salicylate demonstrated a positive mediating effect (12.5%) in the association between tea consumption and CRF risk, while 3-methyl catechol sulfate (-25.70%), glutarate (C5-DC) (-14.60%), and X-23,655 (-18.7%) showed negative mediating effects. In the alcohol consumption-CRF pathway, the ornithine-to-phosphate ratio exhibited a positive mediating effect, while X-23,655 showed a negative mediating effect.

**Conclusion:**

This study provides evidence for a potential protective association of tea consumption and a potential harmful association of alcohol consumption with CRF risk, partially mediated through specific serum metabolites. These findings contribute new insights into potential CRF prevention strategies and may inform dietary guidelines.

**Supplementary Information:**

The online version contains supplementary material available at 10.1186/s12263-025-00773-w.

## Introduction

Chronic renal failure (CRF), or end-stage renal disease (ESRD), represents the most advanced and severe stage of chronic kidney disease (CKD), impacting around 10% of the global population [1, 2]. While genetic predisposition contributes to the development of renal failure, environmental factors, including diet, physical activity, and lifestyle behaviors are increasingly recognized as pivotal in disease progression [3–5]. Among these, beverage consumption—including water, coffee, alcohol and tea—has garnered attention due to its potential association with metabolic health and renal function [6–9]. However, the causal nature of this association remains uncertain due to confounding factors such as socioeconomic status, diet, and lifestyle [2].

Understanding the impact of beverage consumption on renal function is of growing global interest, especially as kidney disease intersects with other health challenges [10]. Despite extensive epidemiological research, confounding factors such as lifestyle choices and socioeconomic status have made it difficult to determine whether associations between beverages consumption and CKD are causal or merely correlational [6, 11]. Mendelian randomization (MR), a method that uses genetic variants as instrumental variables (IVs) to infer causality, provides an innovative approach to addressing this challenge [12]. By leveraging genetic markers linked to beverages consumption and metabolic traits, MR can offer more robust insights into the potential causal effects of these exposures on renal health.

This study applies a two-sample MR analysis to explore the causal relationships between the consumption of different beverages—specifically water, coffee, tea, and alcohol—and renal function, mediated by metabolic pathways. The goal is to provide conclusive evidence on beverage consumption in the development of CRF, offering important implications for dietary recommendations aimed at preventing CKD and managing its progression.

## Methods

### Research design

**Fig. 1:**
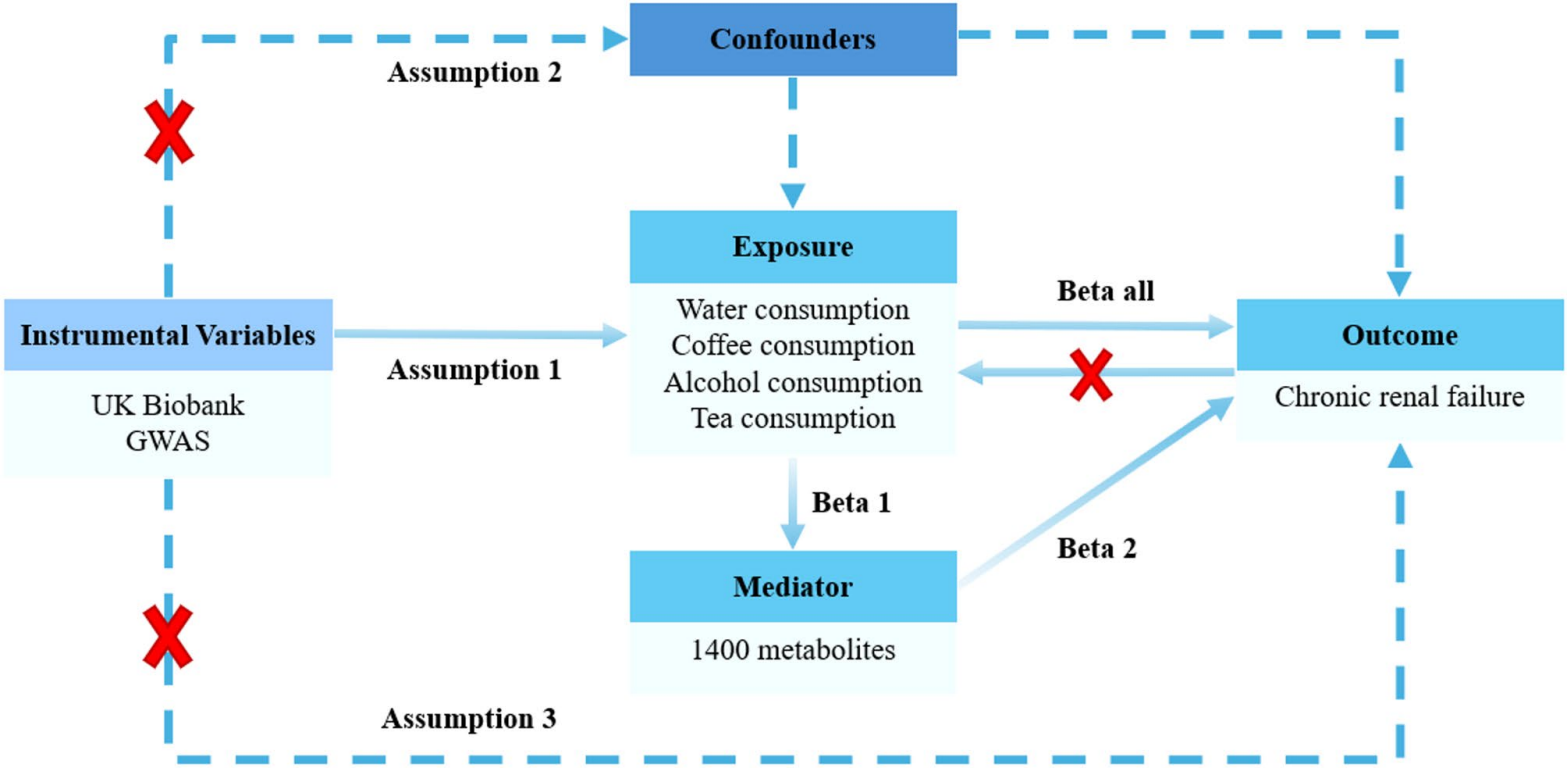
The study design. A two-step MR study of inflammatory cytokines on CRF mediated by metabolites

This research investigated the causal relationship between the consumption of different beverages and CRF using bidirectional MR analysis, while also investigating how circulating metabolites might influence this relationship (Fig. 1). Initially, CRF was defined as the primary outcome, with beverage consumption identified as potential exposures, allowing for a thorough exploration of the causal relationship between these factors. Subsequently, we used 1400 metabolites as potential mediators to screen for those associated with the outcome. Finally, we analyzed the proportions of causal relationships involving selected circulating metabolites, which served as potential mediators, and their links to beverage consumption and CRF.

### Source of data

These manifestations of beverage consumption were extracted from the latest version of the UK Biobank or GWAS, including the presence of water consumption, coffee consumption, alcohol consumption, tea consumption. Metabolites data, including 1,091 metabolites and 309 metabolite ratios, were derived from a GWAS conducted within the Canadian Longitudinal Study on Aging. The relevant GWAS summary statistics can be accessed in the GWAS Catalog under accession numbers GCST90199621 to GCST90201020. The data for the CRF group which GWAS accession number is GCST90018822 included 8, 287 cases and 474, 571 controls (Table 1). These data can be freely accessed in the GWAS database using the identifiers.

**Table 1:** Sources and summaries of genetic data

Phenotype	Sample size	Population	Source	Year
Water consumption	445,799	European	GWAS [13]	2022
Coffee consumption	15,837	Hispanic or Latin American	GWAS [14]	2019
Alcohol consumption	112,117	European	UK Biobank	2017
Tea consumption	434,171	European	GWAS [13]	2022
Metabolites	8299	European	GWAS [15]	2023
Chronic renal failure	8287	European	GWAS [16]	2021

### IVs selection

Single nucleotide polymorphisms (SNPs) were selected as IVs for MR based on the following criteria: (1) They must demonstrate a strong association with the exposure factors. (2) The selected IVs should not be influenced by confounding factors. (3) Selected SNPs should not exhibit pleiotropy, ensuring they influence health outcomes only through the target exposure rather than via other pathways (Fig. 1).

Initially, SNPs strongly associated with beverage consumption, serum metabolites, and CRF (*p* < 5 × 10⁻⁸) were identified through bidirectional causality analyses. We then assessed the linkage disequilibrium, which measures genetic association between each SNP and others, applying the criteria of r² < 0.001 and a distance greater than 10,000 kb to ensure the independence of selected SNPs concerning beverage consumption and metabolites. Furthermore, all palindromic SNPs were removed from the analysis. Finally, we computed the explained variance (R²) and F-statistics to assess the strength of the SNP associations with the risk factors. SNPs with an F-statistic greater than 10 were retained to minimize potential bias from weak instruments. The F-statistic was calculated using the formula: F = R² × (N – 2)/(1 – R²), where R² represents the proportion of variance explained by the SNP, and N denotes the sample size of the exposure data. The R² value itself was estimated using the equation: R² = 2 × (1 – MAF) × MAF × β², where MAF refers to the minor allele frequency, and β represents the effect size of the SNP on the exposure.

###  MR analysis

To determine the overall causal effect of beverage consumption on CRF, we conducted a bidirectional two-sample MR analysis focusing on beverage consumption and its association with CRF. All analyses were carried out using R version 4.3.3(http://www.Rproject.org). Without accounting for heterogeneity and horizontal pleiotropy, we utilized the “Mendelian Randomization” R package to initially assess the causal link between beverage consumption and CRF via the IVW method. Additionally, we applied MR-Egger, weighted median, simple mode, and weighted mode methods to further validate our findings [17–20].

In the two-sample MR analysis, we included serum metabolites that exhibited significant associations with CRF in the mediation analysis. Initially, we evaluated whether these metabolites were significantly related to CRF. If a significant relationship was found, we proceeded with the mediation MR analysis to examine the specific mediation effects of these metabolites. In the first step, we calculated the total causal effect (beta all) of beverage consumption on CRF. Then, we evaluated the causal effects (beta2) of the mediators on CRF and the impact (beta1) of beverage consumption on the metabolites. The mediation analysis was conducted using the “Mendelian Randomization” package, and the mediated proportion was calculated using the formula: Mediated proportion = (beta1 * beta2)/beta all.

### Sensitivity analysis

Cochrane’s Q test was conducted to evaluate the heterogeneity of each SNP, and scatter plots were generated to visualize the consistency of the causal relationship between serum metabolites and CRF characteristics. Furthermore, each SNP was excluded one by one, and the IVW method was applied to the remaining SNPs to assess the potential impact of the excluded variant on the results. PRESSO and Egger regressions were also conducted to check for possible horizontal pleiotropy. Significant outliers identified by MR-PRESSO were subsequently removed. Positive IVW results with p-values ≥ 0.05 across all sensitivity analyses were interpreted as indicative of causal relationships. To reduce Type I errors, False Discovery Rate (FDR) analysis was conducted on the causal relationship between metabolites and CRF.

## Results

### The impact of beverage consumption patterns on CRF

**Fig. 2:**
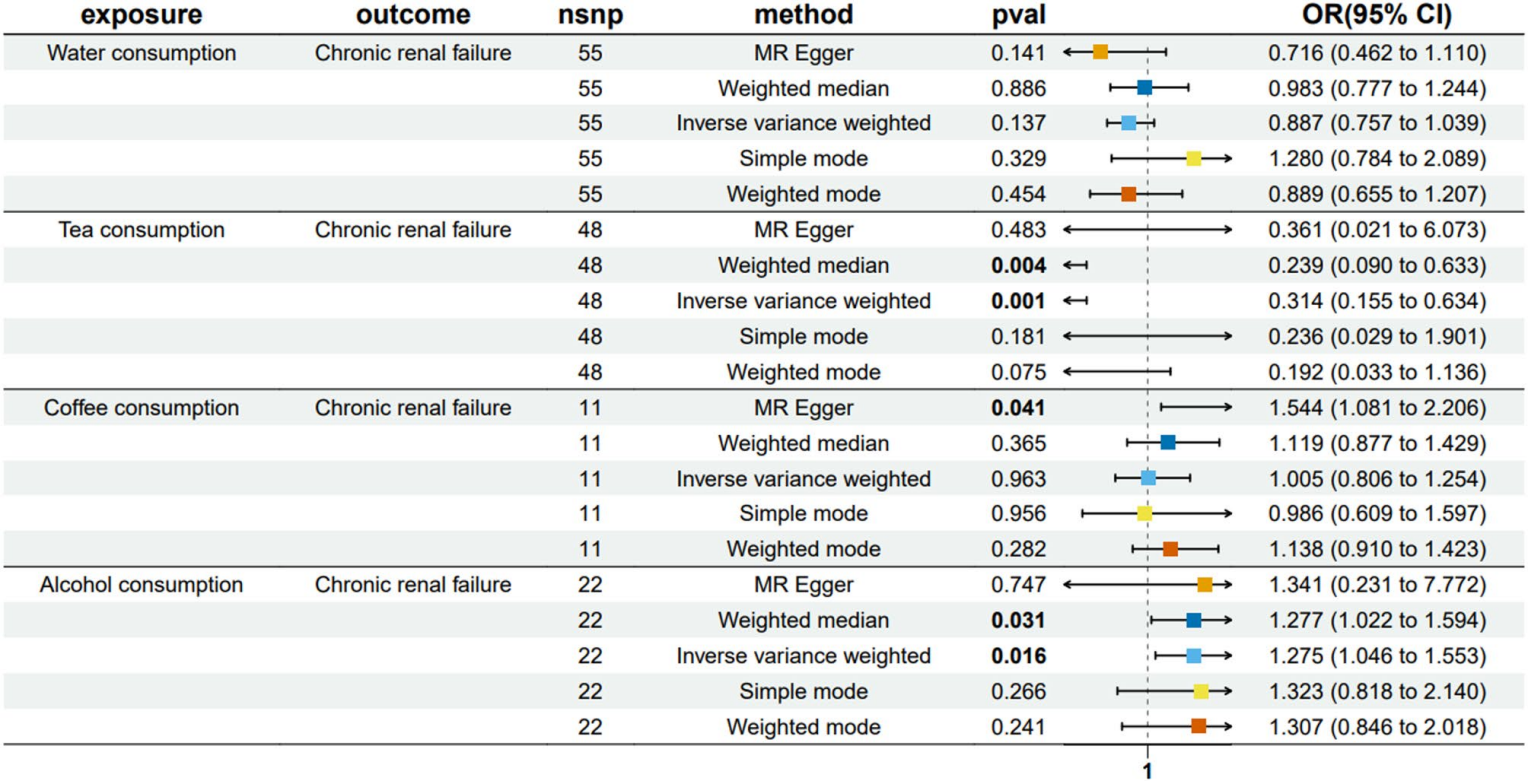
Forest plot of the effects of beverage consumption patterns on CRF

Our research employed a two-directional Mendelian randomization approach to examine correlations between various beverage consumption patterns and CRF. The genetic markers utilized for evaluating the causative links between fluid intake habits and kidney failure likelihood are detailed in Table S1–S5. Figure 2 illustrates the forest plots of the significant estimates from the MR analyses utilizing five different methods. The IVW analysis indicated no significant impact of water or coffee consumption on CRF risk. However, tea consumption was associated with a reduced CRF risk (OR was estimated at 0.314, with a 95% confidence interval between 0.155 and 0.634; *p* = 0.001), whereas alcohol consumption correlated with increased CRF risk (OR estimated at 1.275, 95% CI between 1.046 and 1.553; *p* = 0.016). The weighted median approach similarly confirmed these findings, demonstrating an inverse link between tea drinking and CRF (OR estimated at 0.239, 95% CI between 0.090 and 0.633; *p* = 0.004) and a positive link between alcohol drinking and CRF risk (OR estimated at 1.277, 95% CI between 1.022 and 1.594; *p* = 0.031). The MR-PRESSO and MR-Egger analyses showed no horizontal pleiotropy between exposure and outcome (Tables S6, S7). Cochran’s Q analysis support the lack of genetic heterogeneity (Table S8). Leave-one-out sensitivity analysis confirmed no single SNP significantly affected the metabolite - CRF association (Figure S1).

### Identifying metabolites associated with CRF

**Fig. 3:**
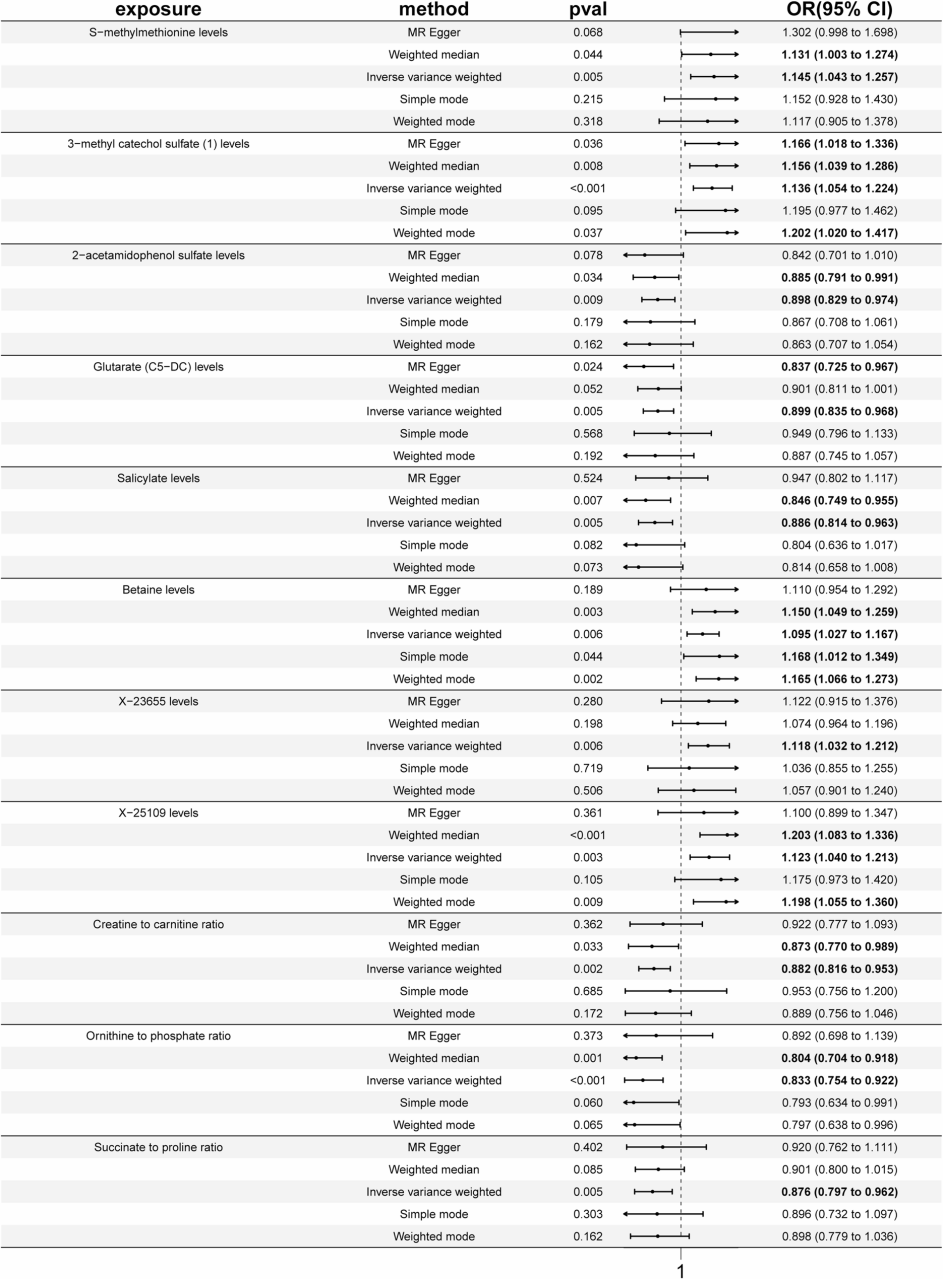
MR analysis of metabolite levels on CRF

The metabolites SNPs are presented in Table S9. To begin with, we screened 67 metabolites or metabolite ratios associated with CRF from a pool of 1,400 metabolites (Table S10). To further enhance statistical differences, we set *p* < 0.01 to narrow the screening scope, identifying 15 related metabolites. Through sensitivity analysis, four of these were further filtered out, ultimately resulting in 11 metabolites significantly associated with CRF (Table S11, S12, S13, Figure S2, Fig. 3).

Positive associations were observed for S-methylmethionine (SMM) levels using the IVW method, with an OR of 1.145 (95% CI: 1.043 to 1.257, *p* = 0.005), and the weighted median method, which showed an OR of 1.131 (95% CI: 1.003 to 1.274, *p* = 0.044). Similarly, 3-methyl catechol sulfate (MCS) levels exhibited positive associations across the IVW method, with an OR of 1.136 (95% CI: 1.054 to 1.224, *p* < 0.001), the weighted median method, where the OR was 1.156 (95% CI: 1.039 to 1.286, *p* = 0.008), and the weighted mode method, which resulted in an OR of 1.202 (95% CI: 1.020 to 1.417, *p* = 0.037). Positive associations for betaine levels were found using the IVW method, with an OR of 1.095 (95% CI: 1.027 to 1.167, *p* = 0.006), and the weighted median method, where the OR was 1.150 (95% CI: 1.049 to 1.259, *p* = 0.003).

In contrast, negative associations were observed for glutarate (C5-DC) levels, with significant findings from the IVW method, showing an OR of 0.899 (95% CI: 0.835 to 0.968, *p* = 0.005), and the MR Egger method, where the OR was 0.837 (95% CI: 0.725 to 0.967, *p* = 0.024). Tea consumption was inversely linked to salicylate levels according to the weighted median method, which indicated an OR of 0.846 (95% CI: 0.749 to 0.955, *p* = 0.005), as well as the IVW method, with an OR of 0.886 (95% CI: 0.814 to 0.963, *p* = 0.007). Negative associations were found for creatine to carnitine ratio (C-C ratio) levels using the IVW method, where the OR was 0.882 (95% CI: 0.816 to 0.953, *p* = 0.002), and the weighted median method, which showed an OR of 0.873 (95% CI: 0.770 to 0.989, *p* = 0.033). 2-acetamidophenol sulfate levels also demonstrated positive associations with tea consumption through the IVW method, where the OR was 0.898 (95% CI: 0.829 to 0.974, *p* = 0.009), as well as the weighted mode method, with an OR of 0.885 (95% CI: 0.791 to 0.991, *p* = 0.034). Similarly, ornithine to phosphate ratio (O-P ratio) and succinate to proline ratio(S-P ratio) levels showed consistent negative associations across methods, suggesting that tea consumption may have protective effects by reducing the levels of these metabolites.

### Mediating effects of metabolites on alcohol consumption - CRF risk

**Fig. 4:**
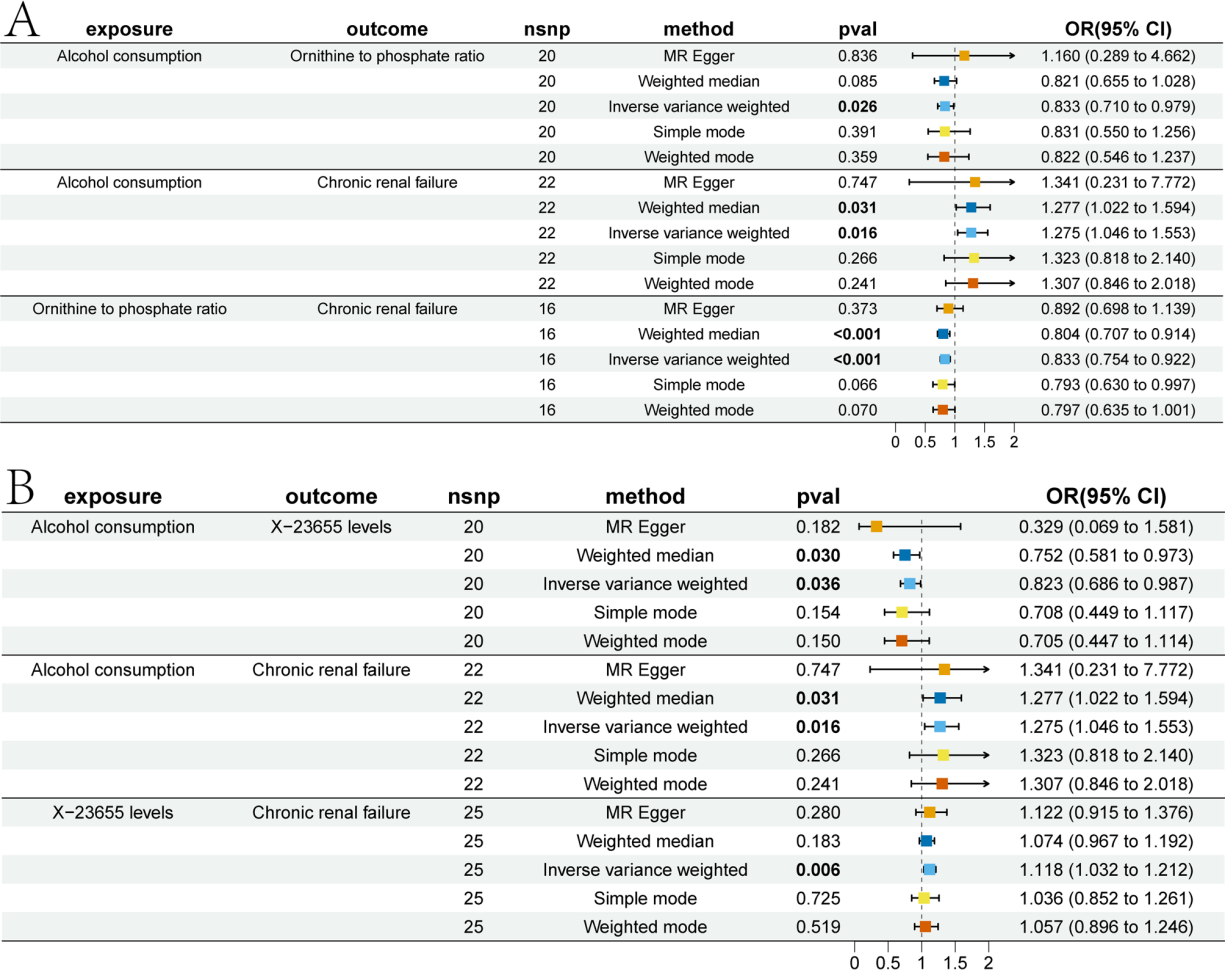
Forest plots depicting the association between alcohol with metabolites

We first analyzed the causal relationship between alcohol consumption and metabolites, and identified two metabolites with causal relationships (Fig. 4). Alcohol consumption mainly exerts its damaging effect on CRF by decreasing O-P ratio levels and X-23,655 levels. Alcohol consumption was negatively associated with O-P ratio levels, with an OR of 0.833, 95% CI: 0.710 to 0.979, and *p* = 0.026 according to the IVW method. For the association between O-P ratio levels and CRF, the weighted median method showed a significant inverse relationship, with an OR of 0.804, 95% CI: 0.707 to 0.914, and *p* < 0.001, while the IVW method also indicated a reverse relationship, with an OR of 0.833, 95% CI: 0.754 to 0.922, and *p* < 0.001. In contrast, X-23,655 was positively associated with CRF, a result supported by multiple methods, including the IVW method, which showed an OR of 1.118, 95% CI: 1.032 to 1.212, and *p* = 0.006. Alcohol consumption was found to have an adverse effect on X-23,655 levels in both the weighted median method, with an OR of 0.752, 95% CI: 0.581 to 0.973, and *p* = 0.030, and the IVW method, with an OR of 0.823, 95% CI: 0.686 to 0.987, and *p* = 0.036. Sensitivity analysis confirmed the robustness of the study results (Tables S14, Figure S3).

### Mediating effects of metabolites on tea Consumption - CRF risk

**Fig. 5:**
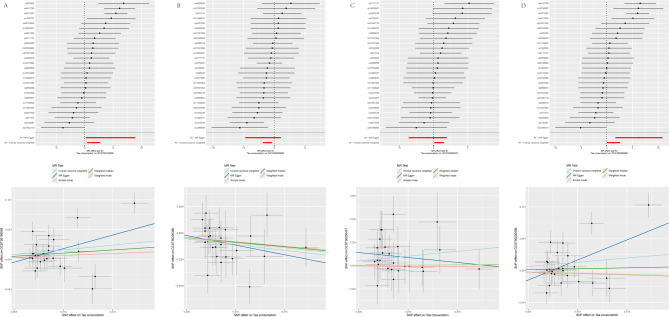
The up panel displays a forest plot of the effect sizes of tea consumption on metabolites, while the down panel provides a scatter plot comparing the effects of SNPs on tea consumption and metabolites

**Fig. 6:**
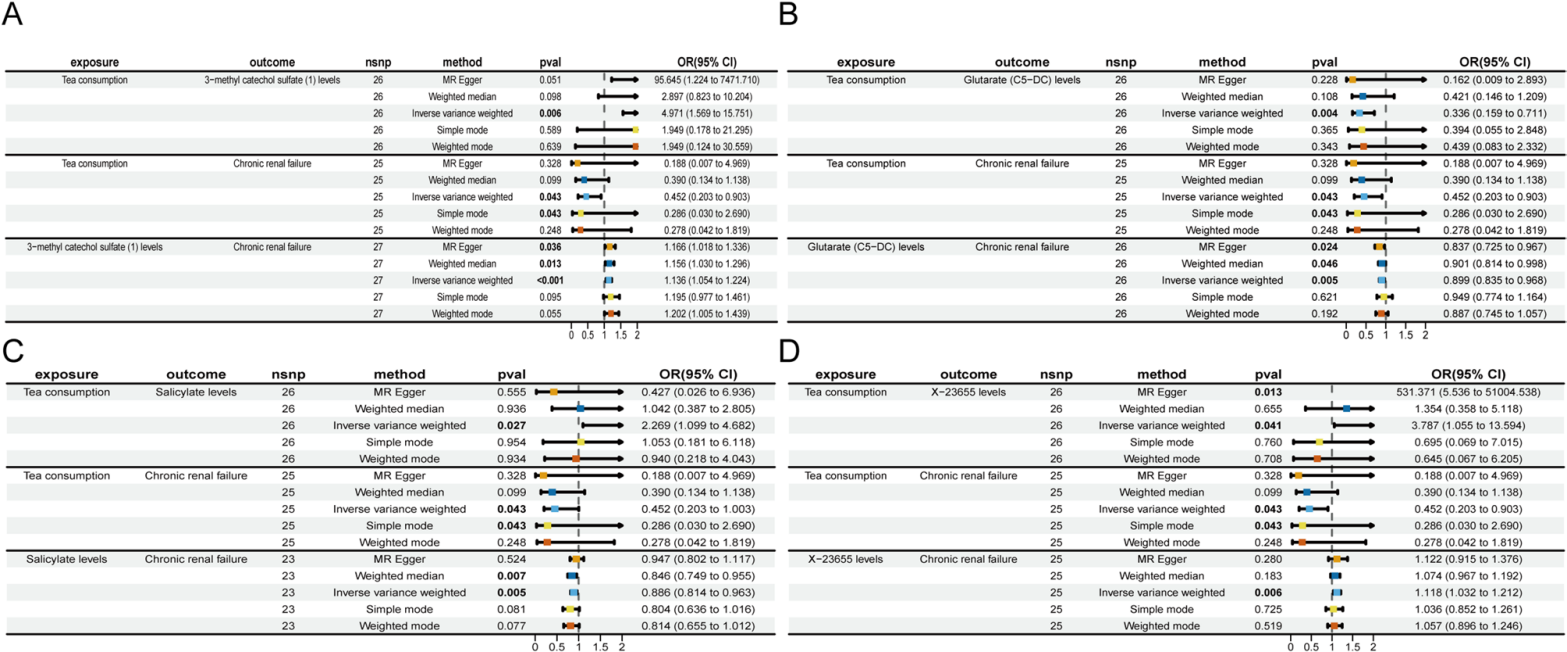
Forest plots depicting the association between tea consumption with metabolites

We then analyzed the causal relationship between tea consumption and metabolites, and identified four metabolites with causal relationships (Figs. 5 and 6). Although other methods did not show statistical significance, the IVW method demonstrated these significant associations. Tea consumption was strongly and positively associated with MCS levels, with an OR of 4.971, 95% CI: 1.569 to 15.751, and *p* = 0.006. Similarly, it was positively associated with salicylate levels, with an OR of 2.269, 95% CI: 1.099 to 4.682, and *p* = 0.027. A positive association was also observed with X-23,655 levels, with an OR of 3.787, 95% CI: 1.055 to 13.594, and *p* = 0.041. In contrast, tea consumption was negatively associated with glutarate (C5-DC) levels, with an OR of 0.336, 95% CI: 0.159 to 0.711, and *p* = 0.004.

Tea consumption mainly exerts its protective effect on CRF by increasing salicylate levels. The IVW method indicated a significant inverse association, with an OR of 0.452, 95% CI: 0.203 to 0.999, and *p* = 0.043, suggesting that tea consumption might reduce the risk of CRF. Additionally, the simple mode method also showed a significant inverse association, with an OR of 0.286, 95% CI: 0.030 to 2.690, and *p* = 0.043. For the association between salicylate levels and CRF, the weighted median method showed a significant inverse association, with an OR of 0.846, 95% CI: 0.749 to 0.955, and *p* = 0.007, while the IVW method also indicated a protective effect, with an OR of 0.886, 95% CI: 0.814 to 0.963, and *p* = 0.043. These findings suggest that both tea consumption and salicylate levels may have protective roles in preventing CRF.

Additional analyses of pleiotropy, heterogeneity, and leave-on-out analysis suggest that the observed associations between tea consumption and metabolites are unlikely to be influenced by confounding factors (Tables S15, Figure S4).

### Mediated MR analysis

We analyzed the causal relationships of tea consumption and alcohol consumption on related metabolites and their impact on CRF before, and then we tried to calculate the metabolites mediation in it (Table 2). In the tea consumption-CRF pathway, MCS (−25.70%), glutarate (C5-DC) (−14.60%), and X-23,655 (−18.7%) all exhibit suppressive mediation effects (negative mediation proportions), indicating these metabolites counteract part of tea’s overall protective association with CRF. This suggests these metabolites may operate through competitive biological mechanisms that work in opposition to tea’s primary protective pathways. Conversely, salicylate demonstrates consistent mediation, accounting for 12.5% of tea’s protective association with CRF risk, suggesting it functions as part of tea’s beneficial metabolic pathway. In the alcohol consumption - CRF pathway, the O-P ratio exhibits a positive mediating effect, while X-23,655 shows a negative mediating effect. Overall, metabolites play a detrimental role in the effect of beverage consumption on CRF.

**Table 2:** Mediated effect of tea and alcohol consumption on CRF via metabolites

Pathway	Beta.1	Beta.2	Beta.all	Mediated effect	Mediated proportion(%)
Alcohol consumption - O/P ratio - CRF	−0.1822	−0.1823	0.2429	0.0332	13.7%
	(−0.3431, −0.0213)	(−0.2830, −0.0816)	(0.0453, 0.4405)	(−0.001, 0.068)	(−0.562%, 27.9%)
Alcohol consumption - X−23,655 - CRF	−0.1948	0.1119	0.2429	−0.0218	−8.97%
	(−0.3765, −0.0130)	(0.0317, 0.1920)	(0.0453, 0.4405)	(−0.058, 0.015)	(−24%, 6.07%)
Tea consumption−3-methyl catechol sulfate (1) - CRF	1.6036	0.1276	−0.7951	0.205	−25.70%
	(0.4504, 2.7569)	(0.0528, 0.2024)	(−1.5928, 0.0026)	(−1.64, 2.05)	(207%, −258%)
Tea consumption - glutarate (C5-DC) - CRF	−1.0900	−0.1065	−0.7951	0.116	−14.60%
	(−1.8395, −0.3405)	(−0.1804, −0.0325)	(−1.5928, 0.0026)	(−0.701, 0.933)	(88.2%, −117%)
Tea consumption - salicylate - CRF	0.8192	−0.1215	−0.7951	−0.0995	12.5%
	(0.0945, 1.5438)	(−0.2054, −0.0375)	(−1.5928, 0.0026)	(−0.693, 0.494)	(87.2%, −62.2%)
Tea consumption - X−23,655 - CRF	1.3316	0.1119	−0.7951	0.149	−18.7%
	(0.0535, 2.6096)	(0.0317, 0.1920)	(−1.5928, 0.0026)	(−1.55, 1.85)	(195%, −233%)

Beta.all: The causal role of beverage consumption on CRF

Beta1: The causal role of beverage consumption on metabolites

Beta2: The causal role of metabolites on CRF

β(mediated effect) = β(beta1) * β(beta2)

Mediated proportion = β(mediated effect) / β(beta all)

## Discussion

Clinicians often tend to believe that drinking tea and alcohol would increase metabolites in the body, thereby exacerbating the burden on the kidneys. However, our MR study has reached a conclusion that differs from this intuitive assumption. This study provides valuable insights into the potential causal relationships between beverage consumption, metabolites, and the risk of CRF using a MR framework. Our findings demonstrate that specific beverages, particularly tea and alcohol, exhibit different associations against CRF, while certain circulating metabolites, such as 3-methylechatechol sulfate, glutarate(C5-DC), X-23,655, salicylate, betaine, and ornithine may mediate these effects.

The relationship between salicylate and renal function has been explored in various clinical contexts, particularly focusing on its cardiovascular benefits and associated renal risks. Salicylates, primarily in the form of aspirin, have anti-inflammatory and antithrombotic effects, which make them a valuable tool in preventing cardiovascular events. However, their impact on renal function remains complex. Studies suggest that low-dose aspirin may offer protective benefits in patients with CKD by reducing the production of thromboxane A2, a potent vasoconstrictor that plays a crucial role in renal hemodynamics and can exacerbate kidney damage [21]. This is particularly important for patients with conditions such as atrial fibrillation or hypertension, where the combination of aspirin with other therapies has been shown to reduce cardiovascular events and slow the deterioration of renal function [21]. The TIPS-3 trial demonstrated that low-dose aspirin could lower cardiovascular events in CKD patients, especially in those with moderate renal impairment [22]. However, concerns remain regarding the increased risk of bleeding in CKD patients due to changes in platelet function [23]. Some studies also suggest that salicylates might accelerate renal decline under certain conditions, particularly when combined with other nephrotoxic agents [24]. The ASCEND and ASPREE trials, which focused on diabetic and elderly populations, revealed inconsistent cardiovascular benefits of aspirin in CKD, with no significant improvement in major cardiovascular events or mortality [25]. Additionally, aspirin use in dialysis patients or those with advanced CKD has been associated with increased adverse effects and minimal clear benefit [26, 27]. Thus, while salicylates may provide cardiovascular protection in early-stage CKD, their use in advanced renal impairment requires careful consideration of the balance between risks and benefits.

Betaine, a natural methyl donor and osmolyte, plays a crucial role in kidney function and shows potential in CKD management. It helps maintain osmotic balance in renal cells, particularly under high osmotic pressure conditions common in CKD patients, thus protecting cells from damage [28]. Betaine also reduces oxidative stress and inflammation in the kidneys, which are two major factors contributing to kidney damage [29, 30]. Additionally, as a methyl donor, betaine lowers homocysteine levels, which are often elevated in CKD and linked to cardiovascular risks and renal damage [31]. By improving lipid metabolism and reducing fatty liver, a condition associated with CKD, betaine further supports renal health [32]. These protective effects make betaine a promising therapeutic agent for managing renal diseases [33].

Previous studies have also found that coffee consumption can lead to changes in MCS levels in human blood, which are xenobiotics involved in benzoate metabolism and may represent potential harmful aspects of coffee on kidney health [34]. Earlier cohort studies suggested that these changes might contribute to impaired kidney function, our MR study confirmed this finding. SMM, also known as vitamin U, is a vitamin-like compound involved in various metabolic processes in the body, including methylation reactions and homocysteine metabolism. Although SMM has antioxidant and lipid-lowering effects that may offer protective benefits for kidney function, our study concluded that SMM is detrimental to kidney function [35]. The direct relationship between SMM and kidney function requires further investigation. Currently, there is also a lack of research on the metabolites X-23,655, 2-acetamidophenol sulfate, O/P ratio, C-C ratio, S-P ratio and glutarate (C5-DC) in the human body, highlighting the need for further exploration of metabolic function on CRF.

Regarding the relationship between coffee consumption and renal function, previous studies have shown conflicting results. A survey study from an East Asian population suggested that coffee consumption is one of the risk factors for CKD in the middle-aged and elderly population [36]. However, more MR analyses indicate that coffee consumption may reduce eGFR and lower the risk of CKD onset [37, 38]. This conclusion has also been confirmed by meta-analyses [39]. Our study found no statistically significant difference in the risk of CRF associated with coffee consumption, which differs from previous research. The discrepancy in results could be attributed to several factors. On one hand, the differences in populations, as our data comes from Hispanic or Latin American groups, which differs from the populations in previous studies. On the other hand, the differences may stem from the nature of the disease itself. CRF is the end stage of CKD, and while coffee consumption may have a protective effect on the development of CKD, it may not influence the progression to CRF. Additionally, we cannot rule out the potential impact of study bias.

MR offers a powerful approach to assess causal relationships between exposures and outcomes by using genetic variants as IVs, minimizing confounding and reverse causation inherent in observational studies and providing robust estimates of causality. Despite the strengths of our study, including the use of a large-scale MR approach primarily with European populations, several limitations warrant consideration: potential limited generalizability to other ethnic groups, possible pleiotropy despite sensitivity analyses, and cautious interpretation of tea’s protective effects against CRF. While tea’s overall protective effect appears to be largely mediated through salicylate, certain compounds like oxalates may impact kidney health differently, and it remains unclear whether tea consumption offers protective benefits for patients already taking aspirin or what the optimal dosage might be [40]. Furthermore, though two-sample MR is widely accepted for studying mediation effects, we must acknowledge its key assumptions: linear and additive relationships between variables, absence of interaction effects, and no residual confounding factors in pathways between metabolites and outcomes—assumptions that may not be fully met in complex biological systems. Future research should consider more sophisticated methods to account for non-linear relationships and interaction effects to validate these findings.

Overall, these findings hold important implications for public health and clinical dietary guidelines aimed at preventing kidney disease. Encouraging the consumption of tea and limiting alcohol intake, while identifying harmful metabolites that contribute to renal dysfunction, may help reduce the global burden of CKD and CRF. Given the intricate relationship between beverage consumption, metabolism, and renal health, additional research is required to confirm these findings across different populations and to delve deeper into the biological mechanisms involved.

## Conclusion

The findings of this study suggest that while water and coffee had no significant impact, alcohol intake was linked to a higher risk of CRF, whereas consuming tea was correlated with a reduced risk of CRF. Tea’s protective effect likely operates through the regulation of salicylate, MCS, and glutarate (C5-DC) levels, whereas alcohol’s influence may be mediated by changes in X-23,655 and the O/P ratio.

## Supplementary Information

Supplementary Material 1.

Supplementary Material 2.

Supplementary Material 3.

Supplementary Material 4.

Supplementary Material 5.

Supplementary Material 6.

Supplementary Material 7.

Supplementary Material 8.

Supplementary Material 9.

Supplementary Material 10.

Supplementary Material 11.

Supplementary Material 12.

Supplementary Material 13.

Supplementary Material 14.

Supplementary Material 15.

Supplementary Material 16.

Supplementary Material 17.

Supplementary Material 18.

Supplementary Material 19.

## Data Availability

Data is provided within the manuscript or supplementary information files.
