# Supplementary figures and images for: The impact of beverage consumption on chronic renal failure risk and the mediation of serum metabolites: based on Mendelian randomization study
Source: Genes Nutr. 2025 Jul 11;20:14. doi: 10.1186/s12263-025-00773-w (PMC12247210; doi:10.1186/s12263-025-00773-w)

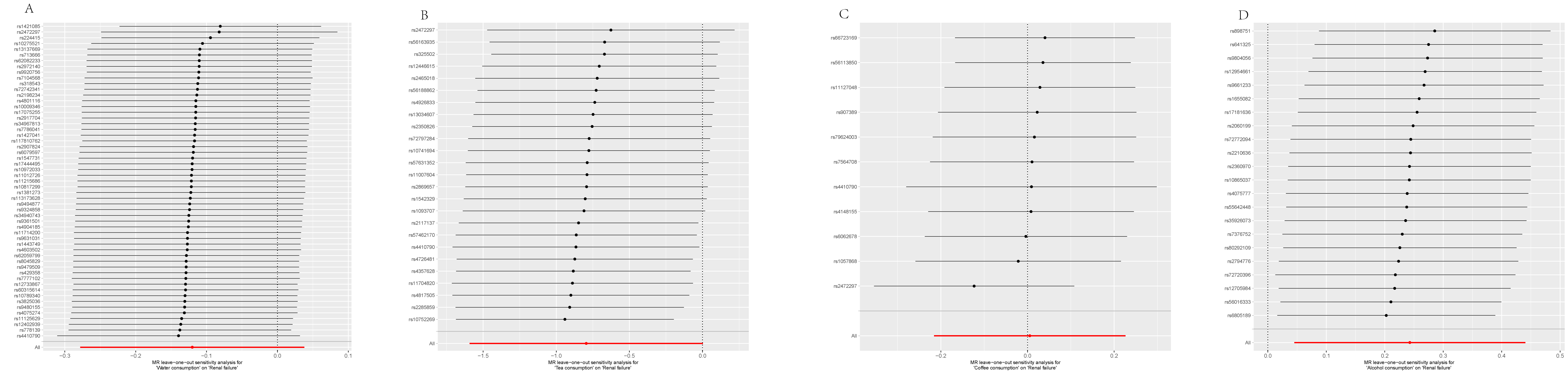

Supplement: Supplementary file 11 — Supplementary Material 11. [file 12263_2025_773_MOESM11_ESM.tif]

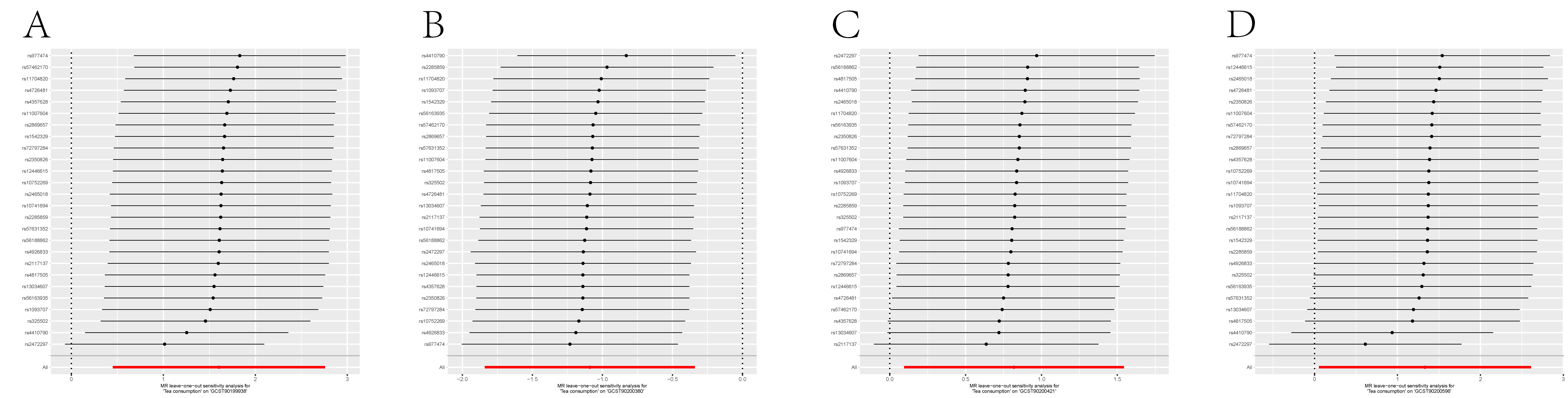

Supplement: Supplementary file 12 — Supplementary Material 12. [file 12263_2025_773_MOESM12_ESM.tif]

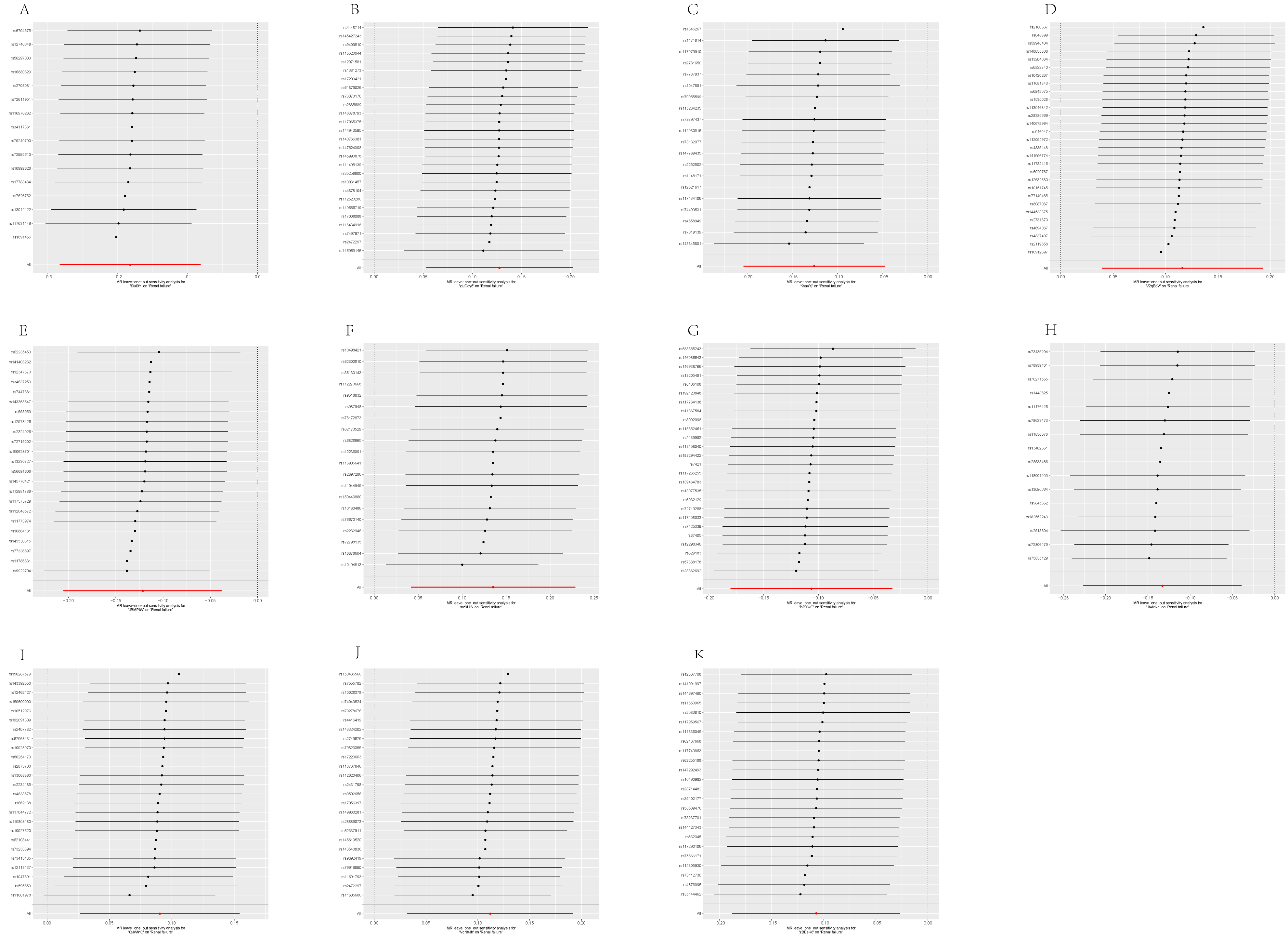

Supplement: Supplementary file 13 — Supplementary Material 13. [file 12263_2025_773_MOESM13_ESM.tif]

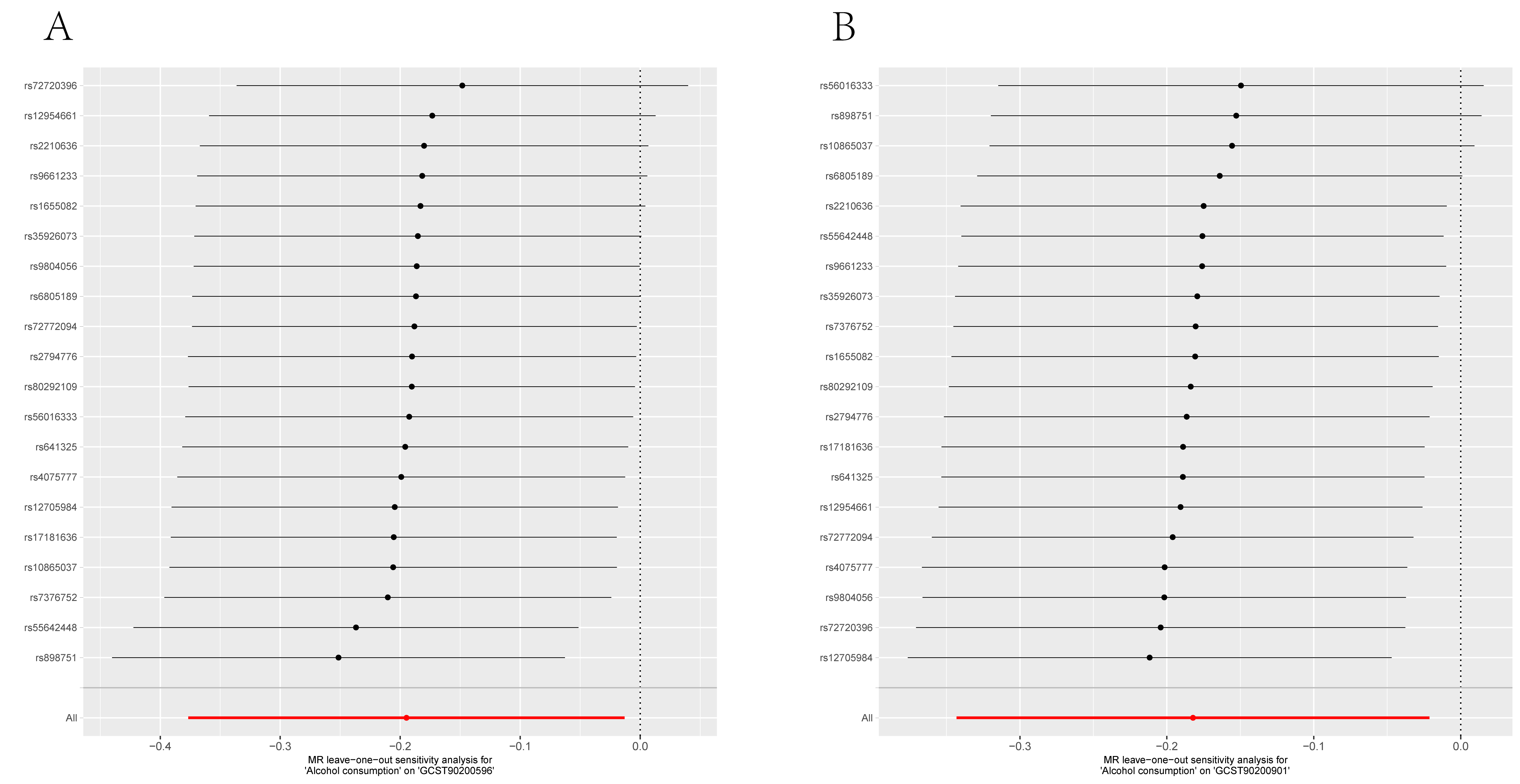

Supplement: Supplementary file 14 — Supplementary Material 14. [file 12263_2025_773_MOESM14_ESM.tif]
